# Uncovering the genetic links of diabetic erectile dysfunction and chronic prostatitis/chronic pelvic pain syndrome

**DOI:** 10.3389/fphys.2023.1096677

**Published:** 2023-02-09

**Authors:** Penghui Yuan, Taotao Sun, Zhengyang Han, Yinwei Chen, Qingjun Meng

**Affiliations:** ^1^ Department of Urology, The First Affiliated Hospital of Zhengzhou University, Zhengzhou, China; ^2^ Department of Urology, Tongji Hospital, Tongji Medical College, Huazhong University of Science and Technology, Wuhan, Hubei, China; ^3^ Department of Ultrasound, The First Affiliated Hospital of Zhengzhou University, Zhengzhou, Henan, China; ^4^ Reproductive Medicine Center, Tongji Hospital, Tongji Medical College, Huazhong University of Science and Technology, Wuhan, Hubei, China

**Keywords:** erectile dysfunction, chronic prostatitis, chronic pelvic pain syndrome, inflammation, biomarker

## Abstract

**Background:** Clinical associations between erectile dysfunction and chronic prostatitis/chronic pelvic pain syndrome (CP/CPPS) have been noticed, but the common pathogenic mechanisms between them remain elusive. The aim of the study was to mine shared genetic alterations between ED and chronic prostatitis/chronic pelvic pain syndrome.

**Method:** Transcriptome data of ED and chronic prostatitis/chronic pelvic pain syndrome-related genes (CPRGs) were retrieved from relevant databases and differentially expressed analysis was used to obtain significant CPRGs. Then function enrichment and interaction analyses were performed to show shared transcriptional signature, including gene ontology and pathway enrichment, the construction of protein-protein interaction (PPI) network, cluster analysis, and co-expression analysis. Hub CPRGs and key cross-link were selected by validating these genes in clinical samples, chronic prostatitis/chronic pelvic pain syndrome and ED-related datasets. Then the miRNA-OSRGs co-regulatory network was predicted and validated. Subpopulation distribution and disease association of hub CPRGs were further identified.

**Result:** Differentially expressed analysis revealed 363 significant CPRGs between ED and chronic prostatitis/chronic pelvic pain syndrome, functioning in inflammatory reaction, oxidative stress, apoptosis, smooth muscle cell proliferation, and extracellular matrix organization. A PPI network containing 245 nodes and 504 interactions was constructed. Module analysis depicted that multicellular organismal process and immune metabolic process were enriched. 17 genes were screened in PPI *via* topological algorithms, and reactive oxygen species as well as interleukin-1 metabolism were regarded as the bridging interactive mechanism. After screening and validation, a hub-CPRG signature consisting of COL1A1, MAPK6, LPL, NFE2L2 and NQO1 were identified and associated miRNA were verified. These miRNAs played an important role in immune and inflammatory response likewise. Finally, NQO1 was identified as a key genetic link between ED and chronic prostatitis/chronic pelvic pain syndrome. It was predominately enriched in corpus cavernosum endothelial cell, and correlated with other male urogenital and immune system diseases tightly.

**Conclusion:** We identified the genetic profiles as well as corresponding regulatory network underlying interaction between ED and chronic prostatitis/chronic pelvic pain syndrome *via* multi-omics analysis. These findings expanded a new understanding for the molecular mechanism of ED with chronic prostatitis/chronic pelvic pain syndrome.

## 1 Introduction

Erectile dysfunction (ED) is a common sexual disorder in men, characterized by the insufficient ability to achieve acceptable sexual performance due to the absence of adequate obtainment or maintenance of penile erectile (Shamloul and Ghanem, 2013). Although it is less likely to threaten men’s life, it troubles quality of life for couples to a great extent. The number of concerned people with ED increases with age, which will reach more than 300 million after 3 years (Ayta et al., 1999; Bacon et al., 2003). Since ED is a pathological process referring to vasculogenic, endocrinological, neurogenic, psychogenic, and other factors, phosphodiesterase type 5 inhibitors (PDE5Is), the first-line remedies tend to exhibit unsatisfactory effects in ED with combined or unknown etiological factors. It is a fact that clarifying the mechanism and distinguishing the specific etiology of ED are the key to the treatment of intricate ED.

Chronic prostatitis/chronic pelvic pain syndrome (CP/CPPS) is the most common type of prostatitis based on the NIH category, which could cause great distress to males from all ages (Khan et al., 2017). It manifests a sense of repeated discomfort or pain in the pelvis accompanied with lower urinary tract symptoms (LUTS) in the absence of infection (Lee et al., 2008). What’s more, its impact on sexual function is usually overlooked (Anderson et al., 2006). Cumulative studies have shown that CP/CPPS is tightly associated with ED (Tran and Shoskes, 2013; Li et al., 2021). Specifically, ED is present in 27%–40.5% of patients with CP/CPPS (Tran and Shoskes, 2013; Li and Kang, 2016). And men with ED had a possibility of previous CP/CPPS diagnosis three times more than control patients (Chung et al., 2012). Although epidemiological link between ED and CP/CPPS has been revealed, it isn’t enough to explain their pathological relationship based on the current studies (Ma et al., 2020). Shoskes et al. (2011) found that men with CP/CPPS tend to have evidence of greater endothelial dysfunction and arterial stiffness. Another study noted that patients suffering from CP/CPPS had higher serum levels of androstenedione and testosterone (Dimitrakov et al., 2008). Moreover, some growth and inflammatory factors involved in ED also existed in CP/CPPS (Dahiya et al., 1999; Pontari and Ruggieri, 2008). Apart from organic factors, psychological factors propelled the pathological interaction in ED and CPPS (Tran and Shoskes, 2013). Also, as one of PDE5Is, tadalafil was proven to be effective in prostatitis (Hiramatsu et al., 2020). It seems that multidimensional pathomechanism coexists in these two diseases. Therefore, mining the overlapping genetic alternations at the molecular level will broaden our understanding of potential mechanisms in ED and CP/CPPS.

Based on these, in the present study, we explored the genetic interrelationships between ED and CP/CPPS based on cell, tissue and human researches for the 1 time. Besides, significant biomarkers and associated biological pathways were identified and analyzed. Our study would help to clarify the common mechanisms and critical regulators behind the interplay of these two diseases, and guide the treatment in the occurrence of ED and CP/CPPS for further research.

## 2 Materials and methods

### 2.1 Data acquisition

The datasets related to ED and CP/CPPS were searched in the Gene Expression Omnibus (GEO) database (https://www.ncbi.nlm.nih.gov/geo/) with the key words “erectile dysfunction,” “chronic prostatitis” and “chronic pelvic pain syndrome”. After screening, five correlative datasets were obtained. Herein, GSE2457 contained expression profiling of corpus cavernosum in rats with ED and corresponding control group (Sullivan et al., 2005). GSE146078 deposited gene data of cavernous endothelial cells in high-glucose and normal-glucose conditions (Yin et al., 2021). Single-cell transcriptome of the corpus cavernosum and microRNA (miRNA) profiles in ED patients were exacted from GSE206528 (Zhao et al., 2022) and GSE182053 (Xu et al., 2021), respectively. In addition, genes related to CP/CPPS were retrieved in the GeneCards database (https://www.genecards.org/) and validated in GSE159438.

### 2.2 Differentially expressed analysis of CPRGs

Genes related to ED in GSE2457 were preprocessed and normalized first, and calibrated genes were processed by “limma” package (Ritchie et al., 2015) in R software (Version 4.2.1) to obtain differentially expressed genes (DEGs) between ED and control groups with the threshold of *p*-value <0.05. Then, the intersection of DEGs in ED and CP/CPPS in GeneCards was performed to obtain significant CP/CPPS-related genes (CPRGs). These results were presented with heatmap and Venn diagram by “pheatmap” package and EVenn (http://www.ehbio.com/test/venn/) (Chen et al., 2021a).

### 2.3 Functional enrichment analysis

After identification of significant CPRGs, Gene Ontology (GO) and pathway enrichment analyses were performed to reveal the possible biological roles of these genes with the threshold of adjusted *p*-value <0.05 and count >2 in the Database for Annotation, Visualization and Integrated Discovery (DAVID) online tool (http://david.ncifcrf.gov). GO was categorized into three subsections: biological process, cellular component, and molecular function, and pathways were conducted by Kyoto Encyclopedia of Genes and Genomes (KEGG) and Reactome. And the results were visualized by “ggpubr” and “ggplot2” packages, respectively.

### 2.4 PPI network and module analysis

Significant CPRGs were uploaded to the Search Tool for the Retrieval of Interacting Genes (STRING) database (http://string-db.org) to depict the interactive functions. The screening criteria was set as an interaction score >0.7 and abandonment of disconnected nodes. Then a protein-protein interaction (PPI) network was constructed in Cytoscape (https://cytoscape.org/, version 3.7.1). In the present network, the Molecular Complex Detection (MCODE) plugin was utilized to conduct module analysis for representing specific molecular complexes with default values (Bader and Hogue, 2003). Additionally, the functional annotation and enrichment analysis of genes in specific modules were performed in Metascape (http://metascape.org) to capture the relationship of enriched terms. It included GO biological processes, KEGG pathway, Reactome gene sets, CORUM and WikiPathways. Then these terms were collected and grouped into clusters based on their membership similarities. Terms with a *p*-value <0.01, a minimum count of 3, and an enrichment factor >1.5 were regarded as significant.

### 2.5 Significant CPRGs detection and functional interaction

The cytoHubba plugin in Cytoscape was applied to screen hub genes by different topological ranking algorithms including BottleNeck, Degree, DMNC, MCC, MNC, and Stress in the considered network (Chin et al., 2014). The overlapped genes in these six methods were further analyzed in the GeneMANIA tool (https://genemania.org/), which provided co-expression and functional analysis based on co-expression, co-localization and predicted interactions (Warde-Farley et al., 2010).

### 2.6 Identification of hub CPRGs and miRNA analysis

The expressed difference of overlapped genes in cytoHubba between ED and control groups was presented in a boxplot with the Wilcox test. Subsequently, genes with statistical significance were validated in GSE206528 from the Male Health Atlas database (http://www.malehealthatlas.cn/) (Zhao et al., 2022), which contained single cell sequencing profiling of corpus cavernosum from eight ED patients and relevant men with normal erection. Hub CPRGs were identified ultimately after screening by similar expressed trend.

Hub CPRGs-associated miRNAs were predicted in miRWalk database (http://mirwalk.umm.uni-heidelberg.de/) based on the reference databases of miRTarBase, TargetScan and miRDB. The expression profiling of these miRNAs was validated in clinical specimens of 20 ED patients and control groups in GSE182053. Then a miRNA–hub CPRGs regulatory network was constructed in Cytoscape. The biological functions of miRNAs were conducted in the miRNA Enrichment Analysis and Annotation Tool (miEAA 2.0) database with the threshold of *p* < 0.05 (https://ccb-compute2.cs.uni-saarland.de/mieaa2/).

### 2.7 External validation of hub CPRGs

To enhance authenticity, the expression profile of hub CPRGs was validated in CP/CPPS-related gene set of GSE159438 and ED-related cellular gene set of GSE146078 with the threshold of adjusted *p*-value <0.05 by “edegR” package, respectively. The final key genes were regarded as genetic links of ED and CP/CPPS. Then the key genes were analyzed in MHA data to show the distribution of cell clusters. Finally, to identify the association between key genes and diseases, the Comparative Toxicogenomics Database (CTD, http://ctdbase.org/) (Davis et al., 2017) was employed to generate their relevance with the inference score >20.

## 3 Results

### 3.1 Identification of significant CPRGs

**FIGURE 1:**
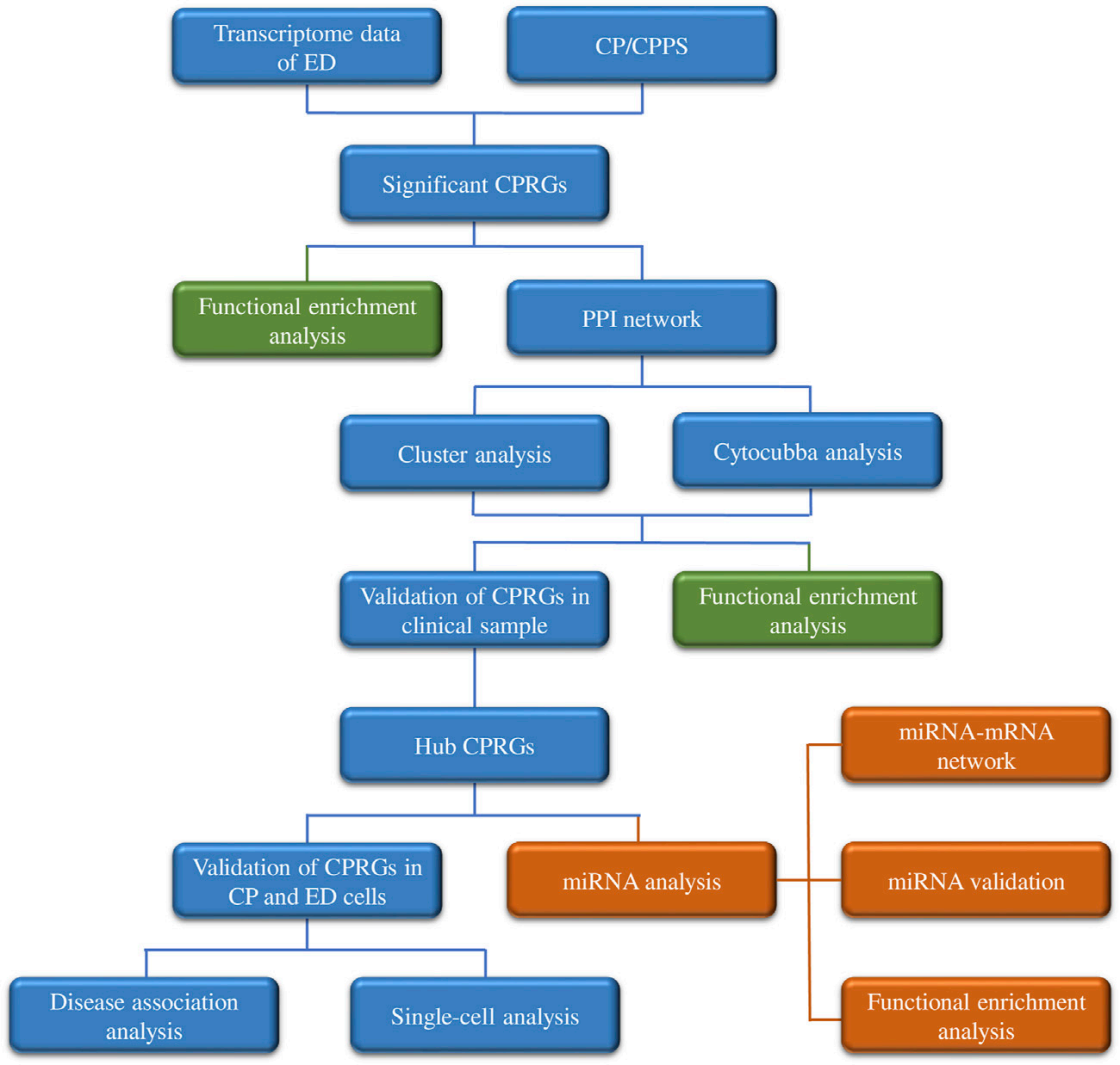
The analytic process of this research. ED, erectile dysfunction; CP, chronic prostatitis; CPPS, chronic pelvic pain syndrome; CPRGs, CP/CPPS-related genes; PPI, protein-protein interaction.

**FIGURE 2:**
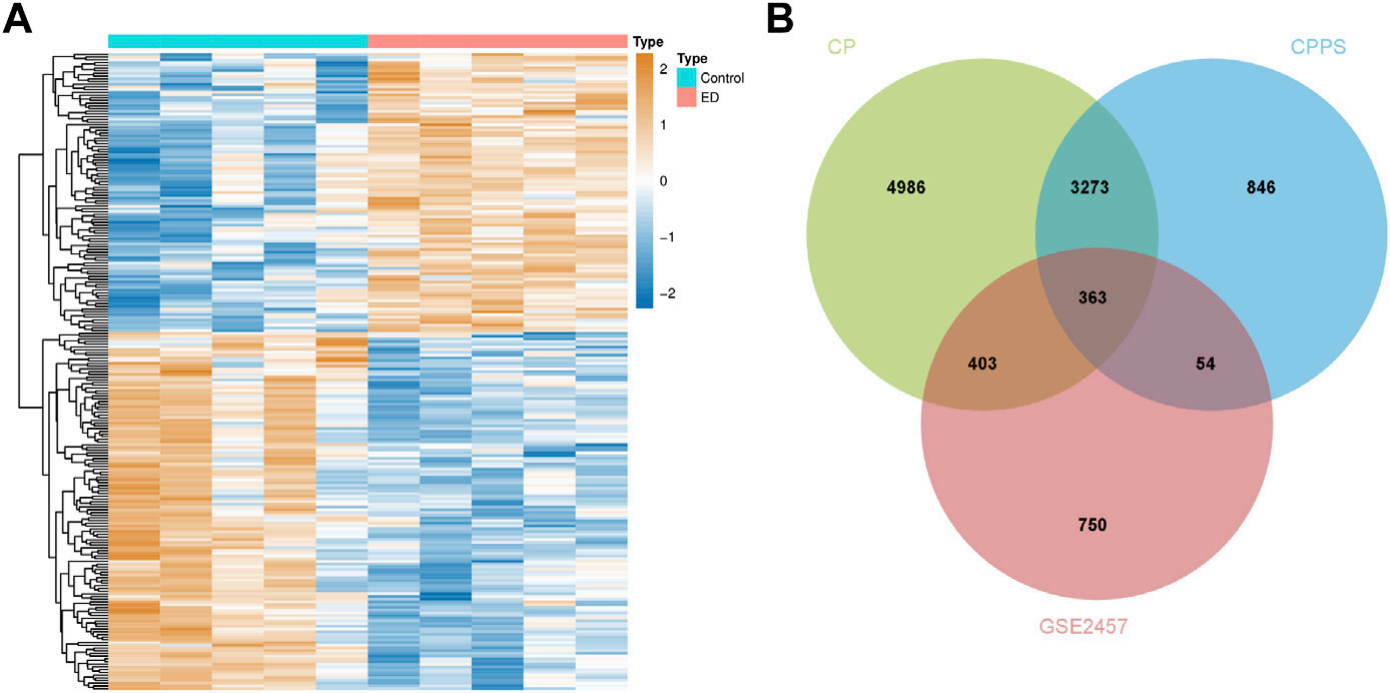
Differentially expressed CPRGs analysis. **(A)** The heatmap of significant CPRGs between the ED and control groups. **(B)** Intersection of DEGs of GSE2457 and CP/CPPS-related gene sets. ED, erectile dysfunction; CP, chronic prostatitis; CPPS, chronic pelvic pain syndrome; CPRGs, CP/CPPS-related genes; DEGs, differentially expressed genes.

The analytic process of this research was depicted in Figure 1. After differentially expressed analysis in GSE2457, a total of 1,570 DEGs were confirmed between ED and control groups. The expression profile of DEGs was presented as a heatmap (Figure 2A). Meanwhile, 9,025 genes related to CP and 4,536 genes related to CPPS were obtained in GeneCards. Then a comparative analysis to determine the overlapped genes was conducted. And 363 significant CPRGs were found consisting of 173 upregulated and 190 downregulated genes for further analysis (Figure 2B).

### 3.2 Functional enrichment analysis of significant CPRGs

**FIGURE 3:**
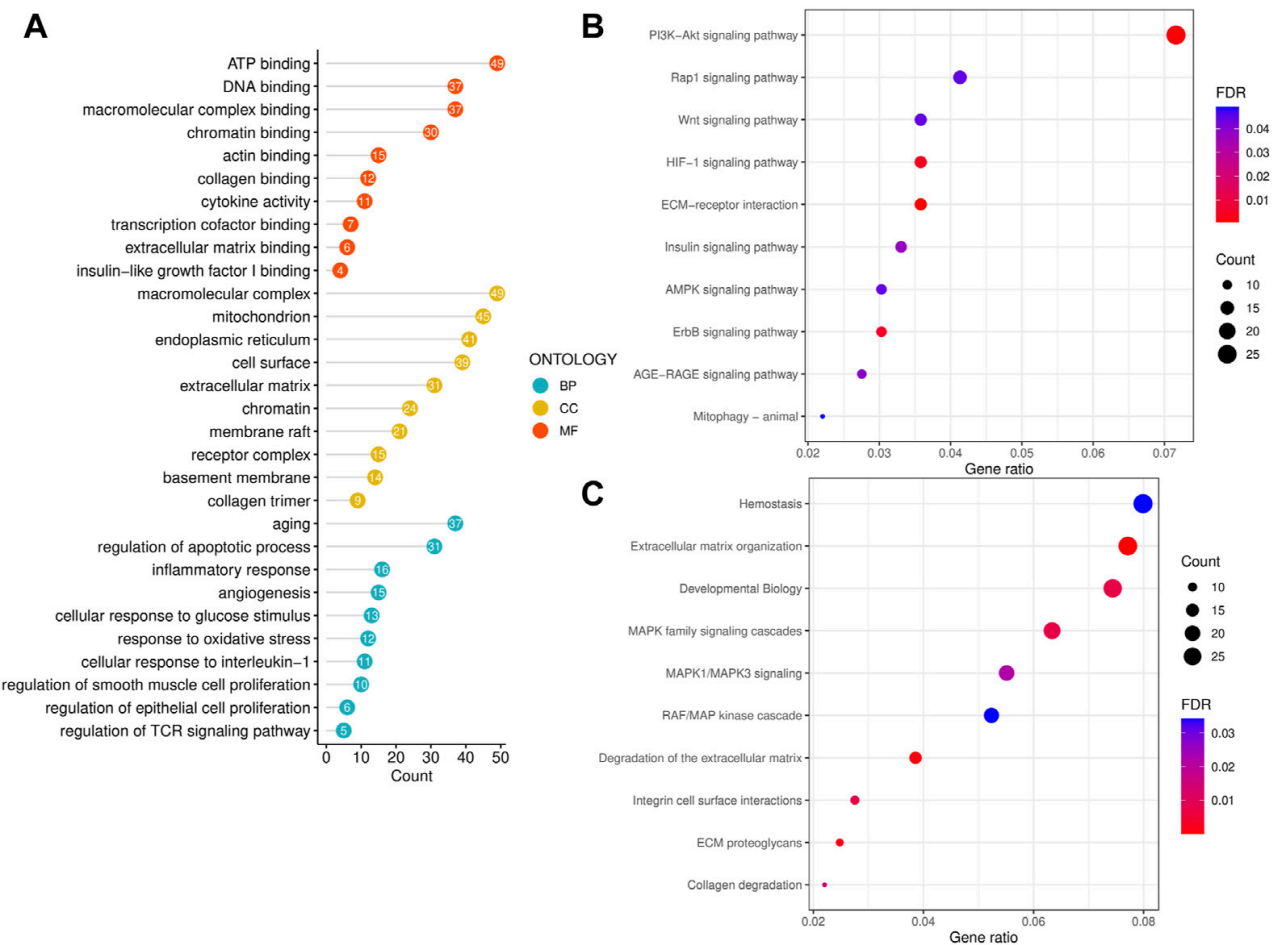
Functional enrichment analysis of significant CPRGs. **(A)** GO enrichment analysis of significant CPRGs. **(B)** KEGG enrichment analysis of significant CPRGs. **(C)** Reactome enrichment analysis of significant CPRGs. CP, chronic prostatitis; CPPS, chronic pelvic pain syndrome; CPRGs, CP/CPPS-related genes; GO, Gene Ontology; BP, biological process; CC, cellular component; MF, molecular function; KEGG, Kyoto Encyclopedia of Genes and Genomes; FDR, false discovery rate.

To reveal the biological functions and associated pathways of significant CPRGs, the function enrichment analysis was performed based on GO, KEGG and Reactome terms (Figure 3A). In GO analysis, biological process revealed significant enrichment of regulation of apoptotic process, smooth muscle cell proliferation, epithelial cell proliferation and TCR signaling pathway, response to interleukin-1, oxidative stress and inflammatory reaction. Cellular component contained extracellular matrix, chromatin, receptor complex, and so on. Molecular function was highly associated with chromatin binding, insulin-like growth factor binding and cytokine activity.

In KEGG analysis, shared pathways contained PI3K-Akt signaling pathway, HIF signaling pathway, and ECM-receptor interaction (Figure 3B). And extracellular matrix organization, integrin cell surface interactions, collagen degradation and MAPK family signaling cascades were observed in Reactome analysis (Figure 3C).

### 3.3 Construction of PPI network and module analysis of significant CPRGs

**FIGURE 4:**
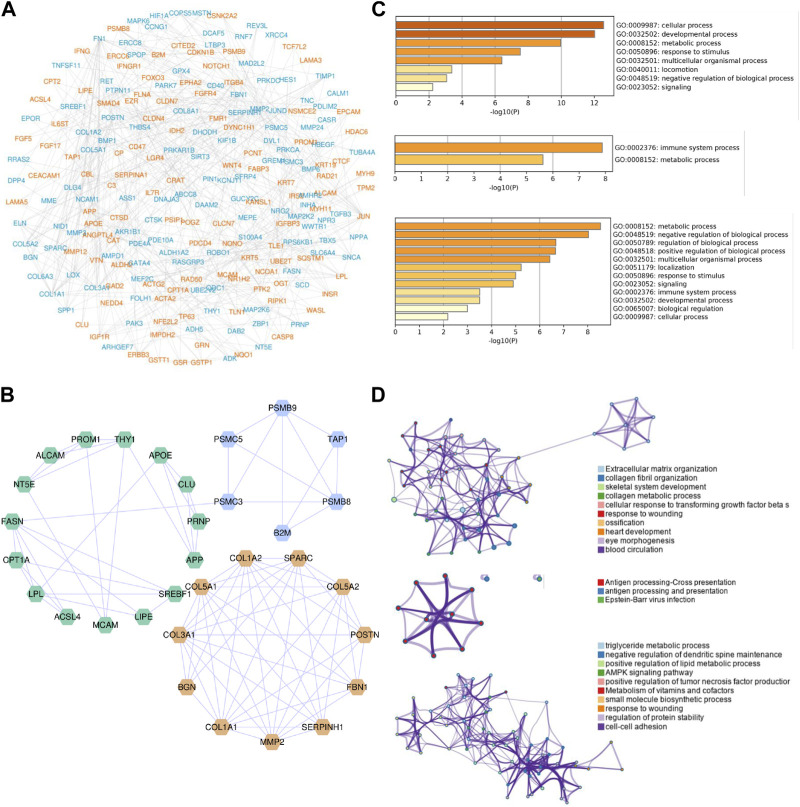
PPI network construction and module analysis of significant CPRGs. **(A)** PPI network of significant CPRGs. **(B)** The top three gene clusters based on module analysis. **(C)** GO enrichment analysis of the top three gene clusters. **(D)** Pathway and process enrichment analysis of the top three gene clusters. For **(A)**, yellow labels represent upregulated CPRGs and blue labels for downregulated CPRGs. For **(B)**, yellow, blue, and green labels represent gene model 1, 2, and 3, respectively. For **(D)**, each node represents an enriched term and is colored by cluster ID. PPI = protein-protein interaction. CP = chronic prostatitis; CPPS, chronic pelvic pain syndrome; CPRGs, CP/CPPS-related genes; GO, Gene Ontology.

To analyze the interplays among these significant CPRGs, a PPI network was constructed. It consisted of 245 nodes and 504 interactions (Figure 4A). Then gene modules in this network were excavated further to reveal differential biological signatures. MCODE analysis presented nine modules, of which the top three modules had a higher density score (Figure 4B). Biological process enrichment analysis showed that genes on module one participated in multicellular organismal process, and collagen metabolic as well as extracellular matrix organization were noted. Module two focused on immune metabolic process, including antigen processing and presentation. Similarly, multidimensional metabolism and immune process were enriched significantly in module three (Figures 4C, D).

### 3.4 Analysis of comprehensive signature of significant CPRGs for further analysis

**FIGURE 5:**
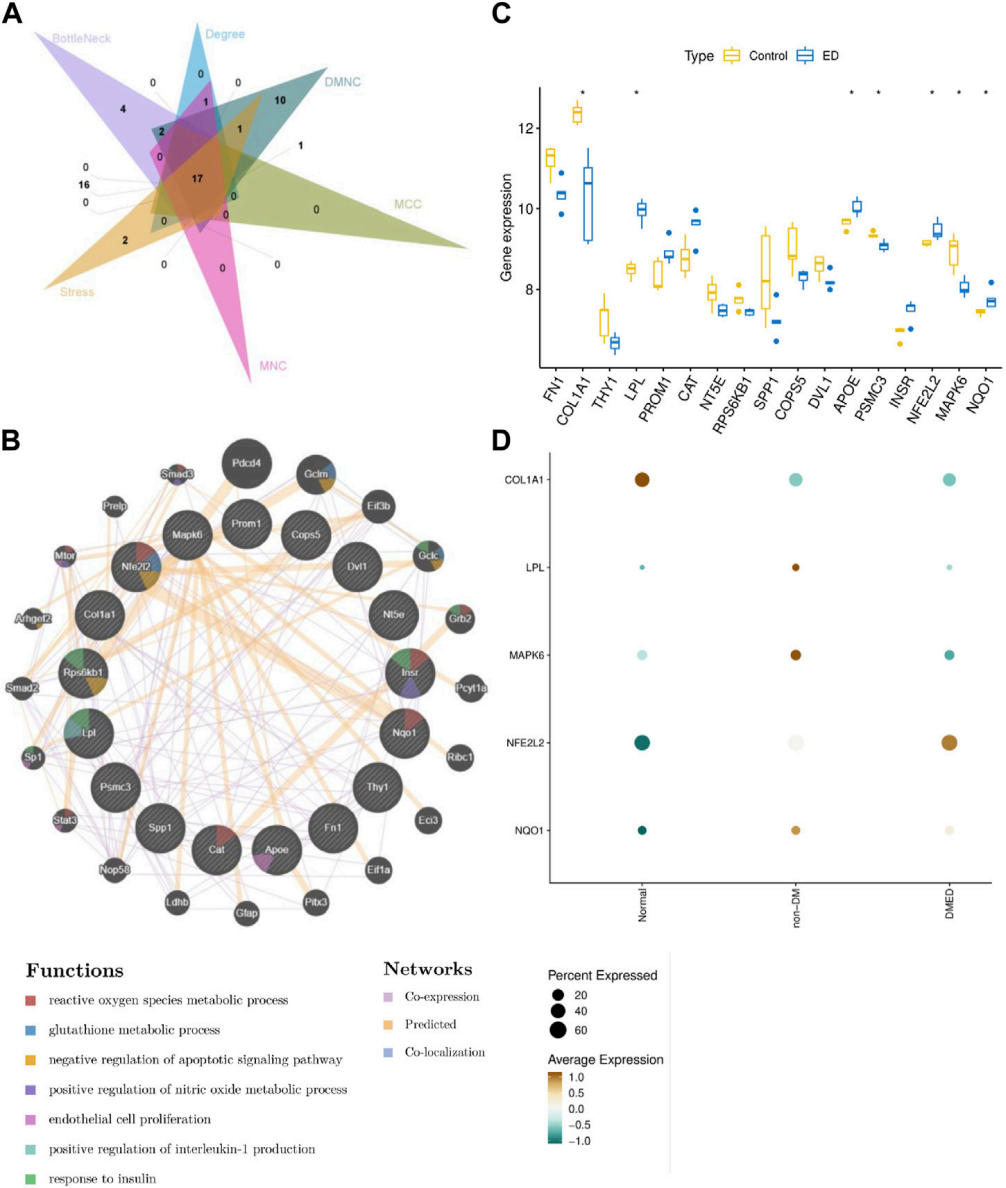
Analysis of comprehensive signature of significant CPRGs **(A)** The intersection of genes *via* six topological ranking algorithms in Cytohubba. **(B)** The interaction information for CPRGs in GeneMANIA. **(C)** The expressed pattern of CPRGs between ED and control groups. **(D)** The expressed pattern of CPRGs in clinical patients with impaired erectile function. For **(B)**, the 20 most frequently changed neighboring genes are shown. The associated neighboring genes are in the outer circle and CPRGs in the inner circle. CP, chronic prostatitis; CPPS, chronic pelvic pain syndrome; CPRGs, CP/CPPS-related genes; ED, erectile dysfunction.

PPI network provided an interactive landscape in these CPRGs. To find the core role in this network, six topological ranking algorithms were utilized to identify the most corresponding components. After comparative analysis, 17 genes exhibited great connectivity (Figure 5A). Furthermore, these genes and 20 their predicted co-expressed genes were displayed in a co-expression network. They were involved in multiple metabolic processes including reactive oxygen species, nitric oxide, interleukin-1, glutathione, and regulation of apoptosis as well as endothelial cell proliferation (Figure 5B).

The expressed pattern of 17 genes in GSE2457 was exhibited in a boxplot. The result showed that seven of 17 genes remained significant differences between ED and control groups (Figure 5C). In the meanwhile, their expressed signatures were also validated in clinical patients with impaired erectile function. Finally, five genes with consistent trends of expression were identified as hub CPRGs (Figure 5D). That’s, COL1A1 and MAPK6 had a lower level of expression in ED group compared with control group. LPL, NFE2L2 and NQO1 showed contrary manifestations.

### 3.5 MiRNA-CPRGs network construction and functional enrichment analysis

**FIGURE 6:**
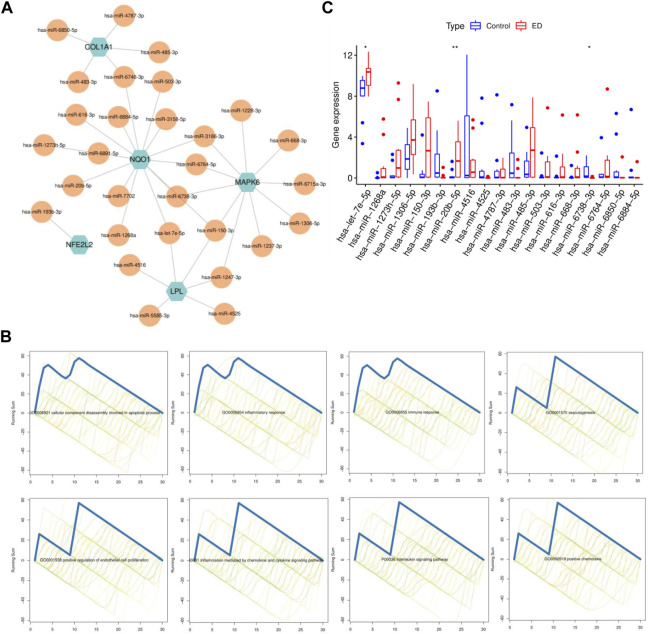
MiRNA-CPRGs network construction and functional enrichment analysis. **(A)** MiRNA-CPRGs co-regulatory network. **(B)** The functional enrichment analysis of miRNAs. **(C)** The expressed pattern of miRNAs between ED and control groups. For **(A)**, green labels represent CPRGs and yellow labels for miRNAs. CP, chronic prostatitis; CPPS, chronic pelvic pain syndrome; CPRGs, CP/CPPS-related genes; ED, erectile dysfunction.

Considering miRNAs could contribute to the process in the interaction of ED and CP/CPPS, miRNAs related to hub CPRGs were predicted first. After validation in clinical specimens, 29 of 862 miRNAs were obtained. Then a co-regulatory network with hub CPRGs as the core components miRNAs as edges was generated (Figure 6A). Consistent with mRNAs, functional enrichment showed that these miRNAs had a tight association with inflammatory response including positive chemotaxis and interleukin signaling pathway, immune response, vasculogenesis (Figure 6B), which suggested a synergetic relationship of CPRGs and miRNAs during the diseases process. Finally, hsa-let-7e-5p, hsa-miR-20b-5p and hsa-miR-6738-3p remained different expression between ED and control groups (Figure 6C).

### 3.6 External validation of hub CPRGs

**FIGURE 7:**
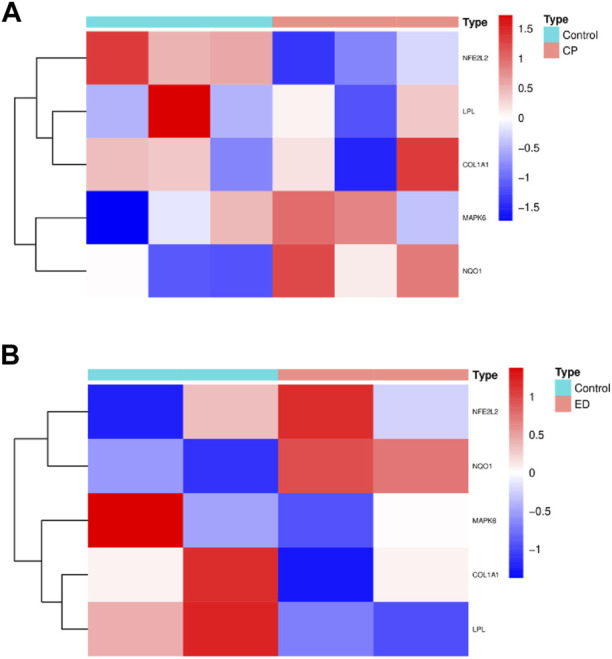
The profiling of hub CPRGs in CP/CPPS **(A)** and ED-related cellular **(B)** gene sets. CP, chronic prostatitis; CPPS, chronic pelvic pain syndrome; CPRGs, CP/CPPS-related genes; ED, erectile dysfunction.

Since CPRGs were screened from ED-related expression matrix and CP/CPPS-related gene sets progressively, to enhance authority and preciseness, hub CPRPs were validated further in external CP/CPPS and ED-related cellular gene sets simultaneously (Figures 7A, B). After performing differentially expressed analysis, overlapped DEGs in the two gene sets were compared with hub CPRGs. Finally, NQO1 was identified. It may serve as a key in the genetic links of ED and CP/CPPS for clinically and pharmacologically oriented research.

### 3.7 Subpopulation distribution and disease association of hub CPRGs

**FIGURE 8:**
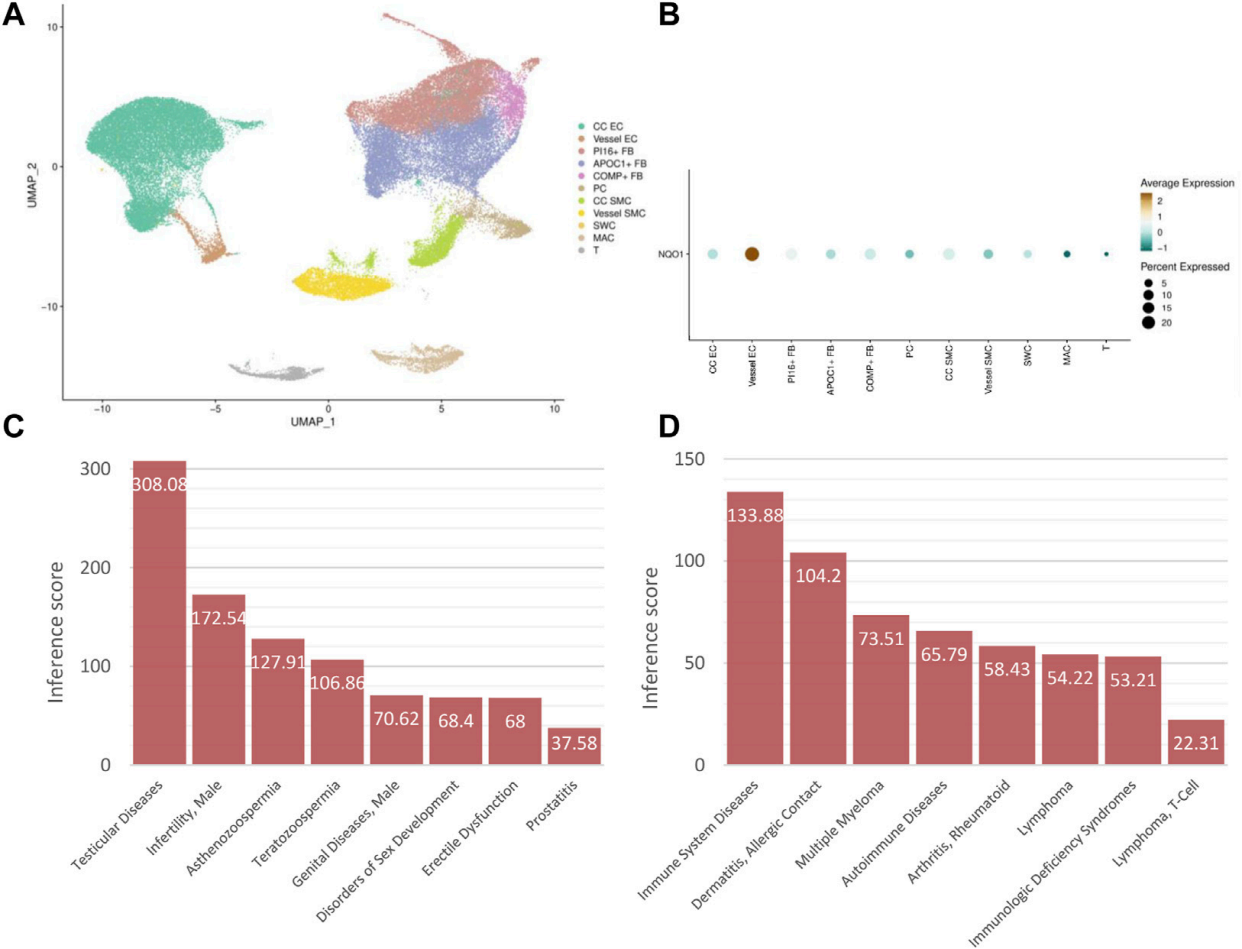
Subpopulation distribution and disease association of hub CPRGs **(A)** UMAP visualization of subpopulations clustering in human corpus cavernosum. **(B)** Expression distribution of NQO1 in ED patients grouped by cell types. **(C)** The association between NQO1 and male urogenital diseases. **(D)** The association between NQO1 and immune system diseases. CP, chronic prostatitis; CPPS, chronic pelvic pain syndrome; CPRGs, CP/CPPS-related genes; ED, erectile dysfunction.

The subpopulation distribution of NQO1 in corpus cavernosum was explored in MHA. The result showed that seven types of cells with 11 subtypes existed in human corpus cavernosum for clustering analysis. They consisted of corpus cavernosum endothelial cell, vessel endothelial cell, PI16-positive fibroblast, APOC1-positive fibroblast, COMP-positive fibroblast, pericyte, corpus cavernosum smooth muscle cell, vessel smooth muscle cell, Schwann cell, macrophage and T cell (Figure 8A). NQO1 was predominately enriched in endothelial cell (Figure 8B).

Considering immune response was enriched in the biological functions of CPRGs and related miRNAs, disease association of NQO1 was utilized in CTD. In male urogenital disease, apart from ED and prostatitis, NOQ1 was associated with testicular and male infertility (Figure 8C). In immune system disease, NOQ1 may be involved in the process of autoimmune diseases and immunological deficiency syndrome (Figure 8D).

## 4 Discussion

High morbidity and prevalence of ED and CP/CPPS in men have been noticed in recent years (Ma et al., 2020). The coexistence of these conditions is attractive and elusive. ED was thought to account for a large proportion among men with prostatitis-like symptoms (Zhang et al., 2015). Also, the history of prostatitis was regarded as an independent risk factor for ED (Ma et al., 2020). There are no studies to investigate the genetic relationship between ED and CP/CPPS. Therefore, this study focused on exploring shared transcriptional alternations, interaction pathways and regulatory network through a systems biology approach. After a multi-omics analysis, hub biomarkers and associated molecular roles were well-mined and validated.

The initial analysis identified 173 upregulated and 190 downregulated shared genes in ED and CP/CPPS. Since both were caused by various etiological factors, to distinguish their heavy interconnection, functional enrichment analysis was conducted first. Obviously, apoptosis, oxidative stress, inflammatory response related to interleukin, and smooth muscle cell proliferation contributed to the predominant functions in these conditions. In a rat model of prostatitis, except for impaired erectile function, CP/CPPS could enhance oxidative stress and calcium imbalance and promote transformation from contractile to synthetic state in corpus cavernosum smooth muscle cells (Wang et al., 2020). Inflammation plays an important role in CP/CPPS and ED. Impaired endothelium could accelerate inflammation in penile vasculature, accompanied by increased inflammatory cytokines like TNF-α and IL-6 (Vlachopoulos et al., 2015; Huang et al., 2019). Hu et al. (Hu et al., 2016) reported that CP/CPPS rendered a systemic inflammatory response in the corpus cavernosum, characterized by increased levels of TNF-α, IL-6 and IL-1β. As we know, oxidative stress and associated apoptosis are crucial pathological phenotypes during the damage of corpus cavernosum endothelial and smooth muscle cells. Elevated production of reactive oxygen species and apoptotic process were noted in the corpus cavernosum of CP/CPPS (Hu et al., 2016). In pathway analysis, PI3K-Akt signaling pathway is a classic process in regulating the activity of endothelial nitric oxide synthase in ED (Li et al., 2017). Similarly, inflammation and oxidative stress could be mediated by PI3K/AKT/FOXO1 pathway in autoimmune prostatitis (Feng et al., 2021). HIF signaling pathway is predominant in hypoxia (Liang et al., 2021). These shared pathways should be considered in the shared pathology of ED and CPPS.

A total of 363 significant CPRGs were heterogeneous gene sets. Cluster analysis in PPI network showed different roles of these genes. It was notable that module two and three focused on immune metabolic process, including 21 genes and 40 interactions. Actually, CP/CPPS was more likely to be considered as an autoimmune disease. Many autoantigens have been identified in the pathogenesis of CP/CPPS (Hou et al., 2009; Liu et al., 2021). Chen et al. (Chen et al., 2021b) showed that the T helper type 1 (Th1) and Th17 cells promoted the progression of CP/CPPS. In detail, Th1 and Th17 immune responses specific to prostate antigens were associated with chronic inflammation of the male genital tract including prostate in patients with CP/CPPS (Motrich et al., 2020). However, apart from increased inflammatory biomarkers, immune process was seldom mentioned in ED. Immune cell infiltration was not observed obviously in diabetic ED (Wang et al., 2022). We speculated that immune response was a particular process in prostatitis complicated with ED.

Ultimately, a 5-gene signature comprising COL1A1, MAPK6, LPL, NFE2L2 and NQO1 were regarded as the hub CPRGs, in which NQO1 may serve as the genetic link in ED and CP/CPPS. COL1A1 encodes the pro-alpha1 chains of type I collagen as a major component of extracellular matrix. It could affect apoptosis by regulating oxidative stress and autophagy in bovine cumulus cells (Fu et al., 2019), and serve as a reliable biomarker in tumors (Ma et al., 2019; Geng et al., 2021). The vital role of COL1A1 accounted for the extracellular matrix-related relationship in ED and CP/CPPS during functional enrichment analysis, and further research should focus on the associated changes. The protein encoded by MAPK6 is a member of the Ser/Thr protein kinase family, which plays an important role in immune and inflammation (Arthur and Ley, 2013). Studies *in vitro* and *in vivo* demonstrated that defects in chemotaxis of monocytes and neutrophils were noticed in MAPK6-deficient cells. MAPK6 was essential to produce several cytokines like IL-8 and activating protein 1 (Bogucka et al., 2020). Therefore, MAPK6 could be thought to construct the inflammatory relationship between ED and CP/CPPS, and worth exploring in depth.

LPL, NFE2L2 and NQO1 showed higher levels of expression in ED group compared with control group. LPL encodes lipoprotein lipase and modifies the regulation of lipid balance in energy homeostasis (Wang et al., 2011). LPL could increase uptake of modified LDL and impair both vascular and endothelial cells, inducing the decrease in eNOS expression. It had a vital impact on blood vessel relaxation (Sullivan et al., 2005). Huo et al. (Huo et al., 2019) found that lncRNA-MIAT downregulation protected erectile function by targeting LPL *via* activating miR-328a-5p in diabetic ED. Although it is not concerned in the available research of prostatitis, it reflected the shared energy metabolism in ED and CP/CPPS. NFE2L2 regulates genes containing antioxidant response elements in their promoters. Pharmaceutical research in ED found that NFE2L2 was a promising target by regulating oxidative stress and inflammatory response in treating ED (Draganski et al., 2018; Pierre et al., 2022). Similarly, in a model of chronic nonbacterial prostatitis, NFE2L2 was involved in the process that tadalafil alleviated inflammation and oxidative stress in RWPE-1 cells (Song et al., 2021). These results propelled the interplay between ED and CP/CPPS.

After comprehensive analysis and validation, a co-regulatory network between hub CPRGs and miRNAs was generated. And hsa-let-7e-5p, hsa-miR-20b-5p as well as hsa-miR-6738-3p remained different expression between ED and control groups. In ED research, Xu et al. (Xu et al., 2021) has revealed that hsa-let-7e-5p was one of the characteristic miRNAs as signature by machine learning method in diabetic ED. Besides, circulating hsa-let-7e-5 could serve as one of the peripheral biomarkers for major depression and bipolar disorders mood disorders (Gecys et al., 2022). And the latter propelled the pathological interaction in ED and CPPS. As for hsa-miR-20b-5p, it was regarded as one of the oncogenic miRNAs in T-cell acute lymphoblastic leukemia by repressing the expression of PTEN and BIM (Drobna et al., 2020). Besides, miR-20b-5p contributed to the dysfunction of aortic smooth muscle cells by targeting MAGI3 in hypertension (Xu and Yu, 2022). The role of hsa-miR-20b-5p in interacting with NOQ1 during the interplay between ED and CP/CPPS should be considered in the further research. Finally, hsa-miR-6738-3p was seldom reported except for primary great saphenous vein varicosities and gastric cancer (Zhang et al., 2018; Fattahi et al., 2021), and its potential functions in ED and CP/CPPS would be analyzed in future experiments.

Our results identified NQO1 as the hub genetic link in ED and CP/CPPS after validation. NQO1 belongs to the NAD(P)H dehydrogenase family and encodes a cytoplasmic 2-electron reductase. Similar to LPL, NQO1 manifests a protective process against inflammatory stimuli and oxidative injury in multiple systems including endothelial and vascular smooth cells (Lee et al., 2021). The finding was consistent with the subpopulation distribution of NQO1 in corpus cavernosum of our results. Zhou et al. (Zhou et al., 2022) pointed out that ED was found in a rat model of hypospadias, accompanied by increased NRF2/Keap-1 ratio and NQO1 expression. Besides, it was regarded as a biomarker against oxidative damage in hydrocortisone-induced ED (Yu et al., 2020). Disease association analysis uncovered the tight correlation of NQO1 and male urogenital disease as well as immune system disease, which showed potential functions in these diseases. It is also significant to explore the specific mechanism of NQO1 in the cross-link of ED and CP/CPPS.

This study could provide new views into the genetic patterns of the shared transcriptional signature in ED and CP/CPPS. However, some limitations were exposed in the present analysis. This study performed relevant molecular excavation based on cell, animal, and human samples with validation, but approach bias *via* the bioinformatics was unavoidable. In addition, due to limited eligible datasets related to ED and CP/CPPS, recapitulating the shared genetic alternations wasn’t persuasive enough. And different types of diabetes were used in the analysis of associated miRNAs. Considering the predominant roles of hub CPRGs in ED ad CP/CPPS, more experimental verification and prospective studies will be conducted in our further research.

## 5 Conclusion

We identified the genetic profiles underlying interaction between ED and CP/CPPS *via* multi-omics analysis. A hub gene signature consisting of COL1A1, MAPK6, LPL, NFE2L2, and NQO1 as well as corresponding regulatory network were well screened and validated. NQO1 was regarded as a key in the genetic links of the two conditions. These findings laid the groundwork for the molecular mechanism of ED with CP/CPPS, and paved the way to fueling the clinically oriented research.

## Data Availability

The datasets presented in this study can be found in online repositories. The names of the repository/repositories and accession number(s) can be found in the article/supplementary material.
